# Capturing the multifactorial nature of ARDS – “Two‐hit” approach to model murine acute lung injury

**DOI:** 10.14814/phy2.13648

**Published:** 2018-03-29

**Authors:** Sandra Hoegl, Nana Burns, Martín Angulo, Daniel Francis, Christopher M. Osborne, Tingting W. Mills, Michael R. Blackburn, Holger K. Eltzschig, Christine U. Vohwinkel

**Affiliations:** ^1^ Organ Protection Program School of Medicine Department of Anesthesiology University of Colorado Aurora Colorado; ^2^ Developmental Lung Biology Cardio Vascular Pulmonary Research Laboratories Division of Pulmonary Sciences and Critical Care Medicine Division of Pediatric Critical Care Departments of Medicine and Pediatrics University of Colorado Anschutz Medical Campus Aurora Colorado; ^3^ Feinberg School of Medicine Division of Pulmonary and Critical Care Northwestern University Chicago Illinois; ^4^ Department of Respiratory Therapy Colorado Children's Hospital Aurora Colorado; ^5^ Department of Biochemistry and Molecular Biology University of Texas Health Science Center Houston Houston Texas; ^6^ Department of Anesthesiology University Hospital of Ludwig‐Maximilians‐University Munich Germany; ^7^ Present address: Department of Anesthesiology University Hospital of Ludwig‐Maximilians‐University Munich Germany; ^8^ Present address: Pathophysiology Department School of Medicine Universidad de la Republica Montevideo Uruguay; ^9^ Present address: Texas Children's Hospital Division of Pediatric Critical Care Baylor College of Medicine Houston Texas; ^10^ Present address: Department of Anesthesiology University of Texas Health Science Center Houston Houston Texas

**Keywords:** Acid aspiration, acute lung injury, ARDS, LPS, ventilator‐induced lung injury

## Abstract

Severe acute respiratory distress syndrome (ARDS) presents typically with an initializing event, followed by the need for mechanical ventilation. Most animal models of ALI are limited by the fact that they focus on a singular cause of acute lung injury (ALI) and therefore fail to mimic the complex, multifactorial pathobiology of ARDS. To better capture this scenario, we provide a comprehensive characterization of models of ALI combining two injuries: intra tracheal (i.t.) instillation of LPS or hypochloric acid (HCl) followed by ventilator‐induced lung injury (VILI). We hypothesized, that mice pretreated with LPS or HCl prior to VILI and thus receiving a (“two‐hit injury”) will sustain a superadditive lung injury when compared to VILI. Mice were allocated to following treatment groups: control with i.t. NaCl, ventilation with low peak inspiratory pressure (PIP), i.t. HCl, i.t. LPS, VILI (high PIP), HCl i.t. followed by VILI and LPS i.t. followed by VILI. Severity of injury was determined by protein content and MPO activity in bronchoalveolar lavage (BAL), the expression of inflammatory cytokines and histopathology. Mice subjected to VILI after HCl or LPS instillation displayed augmented lung injury, compared to singular lung injury. However, mice that received i.t. LPS prior to VILI showed significantly increased inflammatory lung injury compared to animals that underwent i.t. HCl followed by VILI. The two‐hit lung injury models described, resulting in additive but differential acute lung injury recaptures the clinical relevant multifactorial etiology of ALI and could be a valuable tool in translational research.

## Introduction

Acute lung injury (ALI) or acute respiratory distress syndrome (ARDS) are terms to describe a syndrome that, according to the modified definition of the Berlin Consensus conference in 2011(Force et al. 2012), consists of acute onset of mild to severe hypoxemia, pulmonary edema not explained entirely by fluid overload or cardiac disease, and chest X‐ray with bilateral opacities (Eltzschig and Carmeliet 2011; Force et al. 2012). ARDS is common in critically ill patients; cross‐sectional studies indicate that approximately 5% of hospitalized, mechanically ventilated patients fulfill criteria for ARDS (Esteban et al. 2008). With an overall mortality of 40% (Rubenfeld et al. 2005; Villar et al. 2011) and significant long‐term disabilities in ARDS survivors, such as decreased exercise tolerance and increased utilization of health care service (Herridge et al. 2011; Mamary et al. 2011), ARDS contributes significantly to the morbidity and mortality burden of the critically ill. Despite considerable progress made by human studies, which added valuable descriptive information about onset and evolution of ARDS (Matute‐Bello et al. 2008), the fundamental pathomechanisms that are underlying ALI remain poorly understood (Vadasz and Brochard 2012). On the one hand, cell culture systems are only partially able to recapitulate the complex pulmonary microenvironment with interactions between multiple different cells types. On the other hand, the multifaceted clinical presentation of patients makes it difficult to control for variables when investigating a hypothesis. Animal models are an essential tool in testing hypothesis about underlying molecular mechanisms of ALI and can serve as a bridge between the bench and bedside.

At tissue level, ALI in humans is characterized by neutrophilic alveolitis, injury of the alveolar epithelium and endothelium, hyaline membrane formation, and microvascular thrombi. In 2011 the American Thoracic society held an in depth workshop on animal models of experimental lung injury, comprehensively discussing methodology and which models replicate which critical aspects of human disease (Matute‐Bello et al. 2008, 2011). However, most animal models of ALI are limited by the fact that they focus on a singular cause of ALI and therefore fail to capture the complex pathobiology of clinical ALI, which is usually multifactorial. Furthermore, different ALI models have been shown a diverging read‐out of the same pathway (Letsiou et al. 2015), making it difficult to gage the net effect of a certain pathway in a clinical setting. To better model this clinical scenario we provide a comprehensive description of two models of combined acute lung injury. We set out to characterize two‐hit murine models of ALI as translational models of multifactorial ALI. Lung injury was initiated by subacute acid aspiration or intratracheal LPS application followed by ventilator‐induced acute lung injury (VILI) because of the critical role of mechanical ventilation in the care of critically ill patient. We hypothesized, that mice exposed to i.t. LPS or HCl prior to VILI will result in superadditive acute lung injury compared to mice undergoing VILI.

## Materials and Methods

### Materials

Unless otherwise noted, chemicals were obtained from Sigma (St. Louis/MO, USA). Mouse albumin ELISA Quantitation Set was obtained from Bethyl Laboratories (Montgomery, TX, USA). MPO ELISA kit from Hycult Biotech (Plymouth Meeting, PA, USA).

### Animal models of acute lung injury

**Figure 1:**
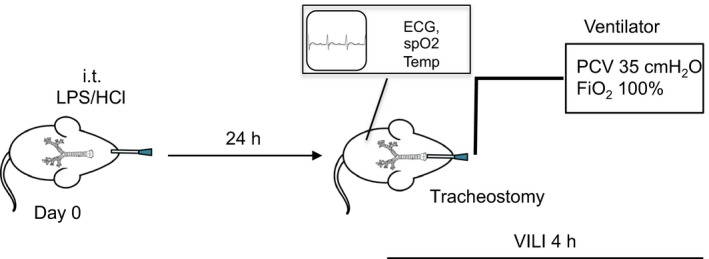
Schematic overview of two‐hit murine ALI models. 8–10 weeks old C57BL/6 mice were anesthetized and 50 *μ*L 0.125 mol/L HCl, respectively, 3.75 *μ*g/g body weight LPS were instilled intratracheally (i.t.). After 24 h mice were ventilated for 4 h with the following parameters: Pressure‐controlled ventilation (PCV), peak pressure 35 cm H_2_O, PEEP 3 cm H_2_O, respiratory rate 80/min, FiO_2_ 1.0.

Animal experiments were approved by the local institutional animal care and use committee (University of Colorado, Anschutz Medical Campus), protocol number B104914(06)1D and B100312(11)1D. All experiments were conducted in 8‐ to 10‐week‐old C57BL/6 mice, weighing 20–25 g. Both male and female animals were used. Animals were monitored for signs of respiratory distress and pain such as overall activity, piloerection, and tachypnea every 12 h according to (Burkholder et al. 2012). Figure 1 depicts a schematic of the experimental protocol.

#### Acid aspiration‐induced lung injury

Acid aspiration model of ALI was described previously (Patel et al. 2012). As Intubation and i.t. instillation can be accomplished in less than 20 sec, isoflurane was used to induce anesthesia. Mice were anesthetized by placing them in a closed Plexiglas box with isoflurane (up to 5% for induction). After anesthesia was achieved, mice were suspended by their incisors from a custom made 45°‐angled mount. Animals were intubated with a 22 G catheter via guide wire using a small animal laryngoscope (Penncentury, Wyndmoor/PA, USA) and 50 *μ*L of 0.125 mol/L hydrochloric acid (HCl) was instilled. Control animals received 50 *μ*L of 0.9 mol/L NaCl.

#### LPS‐induced lung injury

Age‐ (8–12 weeks old) and gender‐matched mice were anesthetized with isoflurane as described above and LPS (*Escherichia coli* 0111:B4, L4391; Sigma, St. Louis, MO, USA; 3.75 *μ*g/g body weight) was administered intratracheally via a 22‐gauge catheter.

#### Ventilator‐induced lung injury

Ventilator‐induced lung injury (VILI) was described previously (Eckle et al. 2008; Hoegl et al. 2015). Briefly, mechanical ventilation utilizing high inspiratory pressure levels (35 cm H_2_O) in pressure‐controlled ventilation mode was used to induce ALI in mice. Anesthesia was achieved with pentobarbital (70 mg/kg i.p. for induction; 20 mg/kg/h for maintenance). The animals were placed on a temperature‐controlled heated table with a rectal thermometer probe connected to a thermal feedback controller to maintain body temperature at 37°C. Tracheotomy and mechanical ventilation were performed as described previously (Eckle et al. 2007; Hoffman et al. 2017). In short, after tracheostomy the catheter inserted into the trachea was connected to a mechanical ventilator (Siemens Servo 900°C, with pediatric tubing) and mice were ventilated for 4 h. The following respiratory parameters were used: Peak inspiratory pressure level 35 cm H_2_O, positive end expiratory pressure (PEEP) of 3 cm H_2_O, respiratory rate of 80/min and inspired oxygen fraction (FiO_2_) of 1. At the end of the experiment, mice were euthanized by exsanguination under deep anesthesia.

#### Combined lung injury

Animals received 50 *μ*L 0.125 mol/L HCl or 3.75 *μ*g/g body weight LPS 24 h prior to ventilation (Fig. 1). The same parameters than in the VILI group were used to ventilate the animals (peak inspiratory pressure level 35 cm H_2_O (15 cm H_2_O in the control group), positive end expiratory pressure (PEEP) of 3 cm H_2_O, inspired oxygen fraction (FiO_2_ of 1)). At the end of the experiment, mice were euthanized by exsanguination under deep anesthesia.

### Sample collection

Bronchoalveolar lavage fluid (BALF) was obtained by lavaging the lungs three times with 1 mL PBS. After centrifugation at 300*g* for 5 min at 4°C, cell‐free BAL was immediately snap‐frozen for subsequent ELISA studies. Pulmonary tissue was flushed with 10 mL saline via the right ventricle, and either snap‐frozen in liquid nitrogen and stored at −80°C or conserved in formalin for histologic analysis.

### Measurement of BALF albumin content, myeloperoxidase assay, and cytokine concentrations

Albumin content of BALF was measured with a mouse albumin ELISA Quantitation Set (Bethyl Laboratories, Montgomery, TX, USA) according to the manufacturer's instructions (Schneider and Issekutz 1996).

Myeloperoxidase (MPO) is rapidly released by activated polymorphonuclear neutrophils, monocytes, and macrophages. MPO concentrations in the BAL were measured with a mouse MPO ELISA kit (Hycult Biotech, Plymouth Meeting, PA, USA) according to manufacturer's instructions. CXCL1 (murine IL‐8 equivalent) and IL‐6 concentrations in the BAL were measured by ELISA using specific antibodies and standards (Duoset, R&D Systems, Minneapolis, MN, USA) according to the manufacturer's instructions.

### Measurement of wet to dry ratio

Wet‐to‐dry ratios were measured as previously described (Eltzschig et al. 2005; Koeppen et al. 2013). In short, after conclusion of experimental treatment lungs were excised en bloc. The weight was obtained immediately after in order to prevent evaporative fluid loss of the tissues. Lungs were then lyophilized for 48 h at 37°C, and dry weight was measured. Wet‐to‐dry ratios were calculated as mg water per mg of dry tissue.

### RNA isolation and real‐time PCR

Total RNA was extracted from lung tissue by Qiazol Reagent (Qiagen Sciences, MD, USA), followed by cDNA synthesis using iScript cDNA Synthesis Kit (Qiagen) according to the manufacturer's instructions. Quantitative reverse transcriptase PCR (Qiagen) was performed to measure relative mRNA levels for various transcripts following manufacturer‘s instructions. The following murine Quantitect Primer Assays were used (Qiagen): *β*‐actin (QT01136772), IL‐6 (QT00098875), CXCL1 (QT00115647). All RT‐PCR assays were standardized relative to *β*‐actin levels and are presented as fold change in mRNA expression relative to controls.

### Lung histology and lung injury scoring

After completion of the experiment the lungs were inflated at 15 cm H_2_O with 4% paraformaldehyde and remained in paraformaldehyde solution till further processing. Dehydration of the formalin‐fixed lungs was achieved by placing them in ethanol gradients. The dehydrated lungs were then embedded in paraffin, sectioned at 5 *μ*m, and stained with H&E (Baker 1962). Assessment of histological lung injury was performed by grading as follows (Ehrentraut et al. 2013): infiltration or aggregation of inflammatory cells in air space or vessel wall: 1, only wall; 2, few cells (one to five cells) in air space; 3, intermediate; 4, severe (air space congested); interstitial congestion and hyaline membrane formation: 1, normal lung; 2, moderate (<25% of lung section); 3, intermediate (25–50% of lung section); 4, severe (>50% of lung section); hemorrhage: 0, absent; 1, present. Six representative images were obtained from each animal and were analyzed blinded to group assignments.

### Stains to assess fibrosis

Lungs were formalin‐fixed, dehydrated, and embedded in paraffin as described above.

#### Masson's trichrome stain

Staining of deparafined and rehydrated 5 *μ*m lung sections with Masson's trichrome stain has been described in detail in (Zhou and Moore 2017) and is a well‐established method in assessing lung fibrosis (Zhao et al. 2002; Liu et al. 2010; Cheresh et al. 2015; Zhou and Moore 2017).

#### Picosirius red stain

In order to specifically assess and quantify collagen deposition, staining with picosirius red was performed. The assay was performed by the UC Cancer Center Histology Core facility as described previously (Puchtler et al. 1973; Junqueira et al. 1979). Collagen deposition was quantified using MetaMorph (Molecular Devices LLC, Sunnyvale, CA) as described previously (Kumar et al. 2017). In short, 10 images of each mouse were acquired at 4× magnification and superimposed on a 17,664‐point grid using MetaMorph software. The same threshold for positive polarization with picrosirius red staining was utilized in all images. The number of positive pixels, which intersected with the grid, divided by the total number of grid points within the area of interest, was calculated as the volume fraction of lung in the region of interest.

### Statistical analysis

Parametric data were compared by one‐way ANOVA with Bonferroni's post hoc test or *t* test where appropriate. Comparison of nonparametric results between groups was performed using the Mann–Whitney U‐test. Values are expressed as mean ± SD. *P*<0.05 was considered statistically significant. For all statistical analysis GraphPad Prism 5.0 software (GraphPad Software, San Diego, CA, USA) was used.

## Results

### Physiology

**Figure 2:**
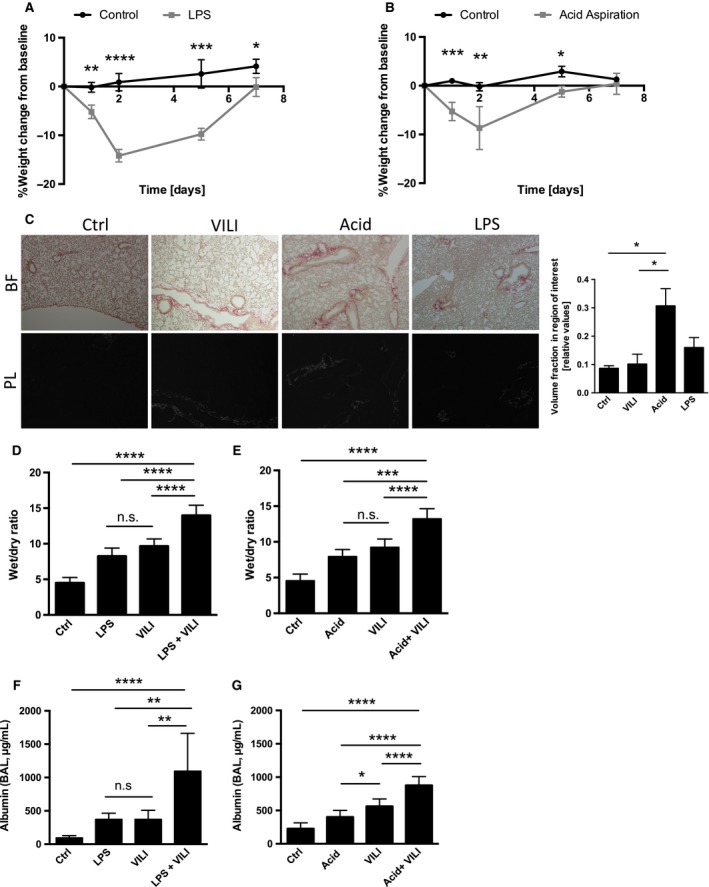
Physiologic changes in two‐hit ALI models. Body weight variation presented as change from baseline line body weight in %. 3.75 *μ*g/g LPS (A), respectively, 50 *μ*L of 0.125 mol/L HCl (B) was instilled i.t., control animals received 50 *μ*L of NaCl. Lung tissues stained with picrosirius red stain (PR) to assess for lung fibrosis. Images were taken either with polarized light (PL) or bright field (BF) (C). Changes in alveolar‐capillary barrier measured by wet‐to‐dry ratio (D, E) and albumin in BAL fluid (F and G). Data are represented as mean ± SD,* n* = 6–7 per time point, n.s. –not significant. *P < 0.05, **P < 0.01, ***P < 0.001, ****P < 0.0001

The animals pretreated with LPS HCl showed significant weight loss up to 15% of their base body weight (Fig. 2A and B) compared to control animals (i.t. instillation of 50 *μ*L NaCl). Concomitantly with the weight loss during the first 48 h post treatment mice in both groups displayed less activity, piloerection and tachypnea which is consistent with clinical signs of acute lung injury in mice (O'Dea et al. 2009; Patel et al. 2012). Both HCl‐ and LPS‐treated animals returned to their pretreatment body weight by day 7. Of note, no mortalities occurred in either groups (except for one animal 2nd to tracheal perforation and subsequent development of a pneumothorax during intubation), which was likely due to the relatively low dose of HCl and LPS. Mice treated with HCl and LPS showed mild fibrosis depicted as volume fraction of lung in the region of interest (in slides stained with picrosirius red under polarized light), mostly around the smaller airways on day 3 with the LPS treated animals showing a trend to less fibrosis than animals treated with HCl (Fig. 2C).

### Alveolo‐capillary barrier function

The integrity of the alveolo‐capillary barrier function was assessed by obtaining the wet/dry ratio in order to determine lung water content and albumin content of the BALF was measured as an indirect method to gauge alveolo‐capillary barrier integrity.

Mice were allocated to following treatment groups: (1) the control group for acid aspiration and LPS instillation (ctrl) received i.t. instillation of NaCl. In preliminary experiments we compared this control group to animals who had received low tidal volume mechanical ventilation (peak inspiratory pressure level 15 cm H_2_O, PEEP of 3 cm H_2_O, FiO_2_ 21% or 100%) and found no difference in histology or mRNA expression of proinflammatory cytokines compared to sham and treated group, that underwent NaCl i.t instillation only (see Fig. S1). (2) i.t. instillation of LPS without ventilation (LPS), (3) Acid aspiration without ventilation (Acid), (4) VILI group without pretreatment with LPS or acid (VILI), (5) i.t. LPS and subsequent VILI 24 h later (LPS+VILI), and (6) i.t. HCL and subsequent VILI 24 h later (Acid + VILI). Both i.t. instillation with LPS and HCl lead to increased wet‐to‐dry ratio (Fig. 2D and E) and albumin content of the BALF (Fig. 2 F and G) indicating alveolo‐capillary barrier dysfunction. Of note, here was a tendency in the mice subjected to VILI induced ALI alone to have increased pulmonary leakage compared to the mice exposed only to i.t. LPS or HCl. Animals that underwent the two‐hit model both with i.t. HCl and LPS instillation followed by VILI had significantly increased alveolo‐capillary barrier dysfunction compared to animals only receiving LPS or HCl (Fig. 2D–G). However, i.t. LPS application prior to VILI had a greater impact on alveolo‐capillary barrier function than i.t. HCl prior to VILI (Fig. 2D–G).

### Lung injury scoring

**Figure 3:**
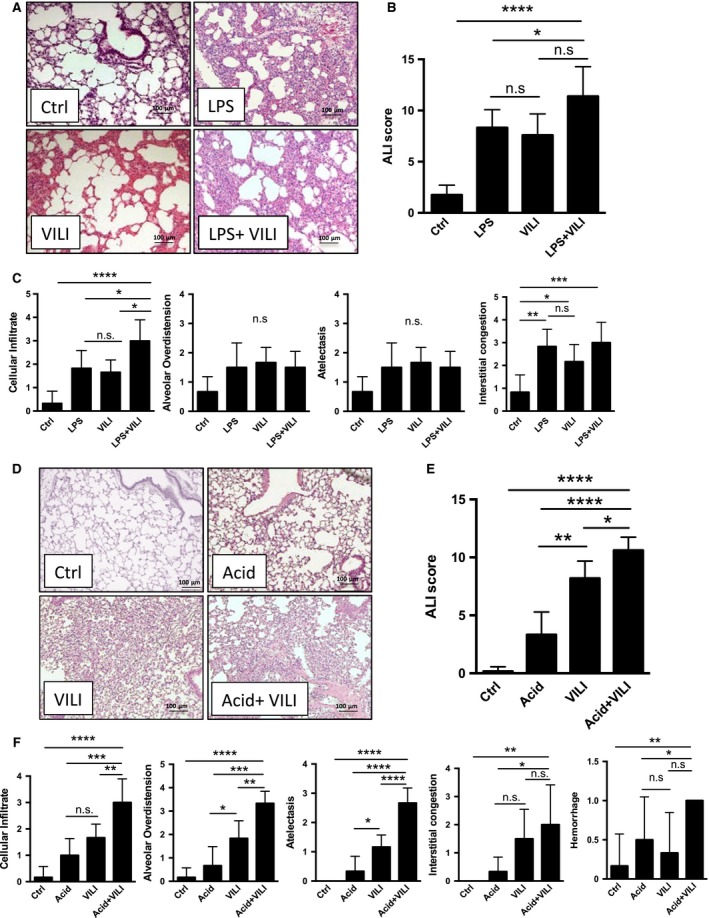
Histologic changes in single and two‐hit ALI models. Panel A and D show representative hematoxylin and eosin (H&E) stained sections of murine lungs after experimental treatment. The cumulative lung injury score (B and E) is a combined score of cellular infiltrate, alveolar over distension, atelectasis, interstitial congestion and hemorrhage, each score on a scale of 0–4 per category (C,F) ± hemorrhage (0/1). Data are represented as mean ± SD,* n* = 6, n.s. –not significant, *P*< 0.05, **P< 0.01, ***P<0.001, ****P<0.0001

Both treatment with LPS and HCl resulted in substantial changes to the lung architecture compared to control animals, that was exacerbated when LPS and HCl instillation was followed by VILI (Fig. 3A–B and Fig. 3D–E). When looking at the individual components of the histology of lung injury, both LPS and HCl exposure resulted in significant cellular infiltration, the alveolar inflammation was exacerbated in both the combined lung injury models (Fig. 3C and F). Furthermore, animals in the LPS group (treated only with LPS or LPS followed by VILI) had a trend toward increased, but not significant alveolar overdistension and atelectasis, whereas acid (alone or in combination with VILI) resulted in clearly increased alveolar overdistension and atelectasis. Animals subjected to i.t. LPS had marked interstitial congestion compared to the animals treated with i.t. HCl which is in line with our findings and that of Patel et al. (2012) showing increased collagen deposits (see Fig. 2C). There was no hemorrhage observed in the LPS only or LPS+VILI‐treated animals in contrast to the acid and acid+ VILI‐treated animals.

### Alveolar inflammation

**Figure 4:**
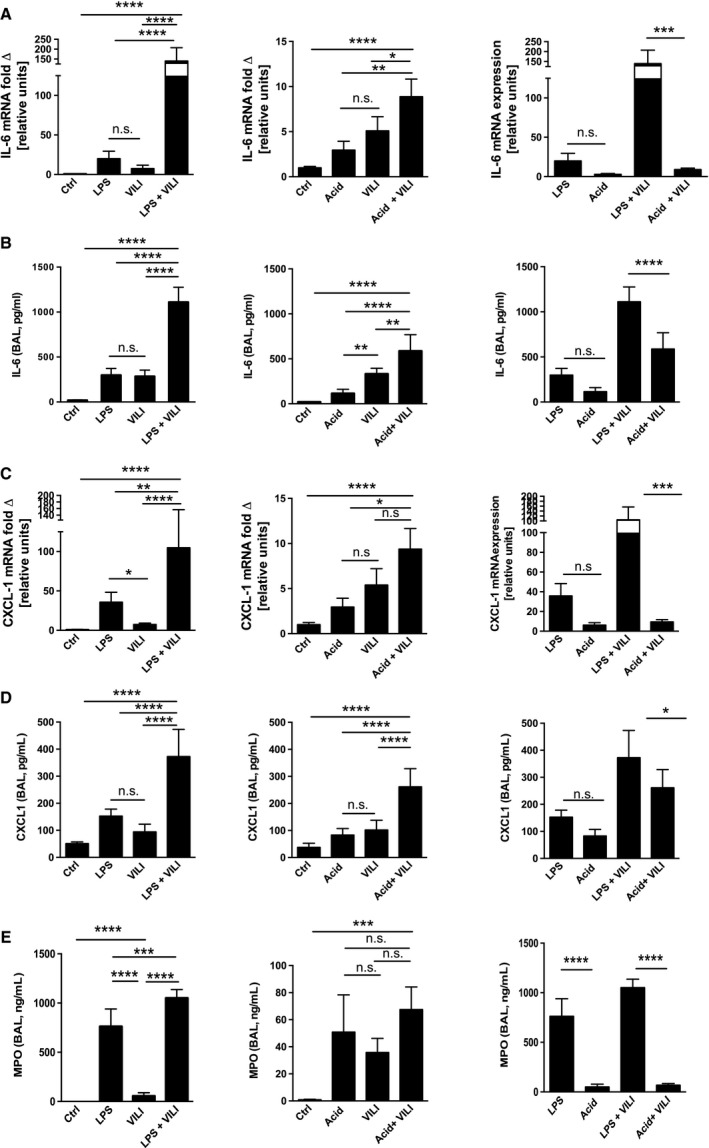
Pro‐inflammatory cytokines in two‐hit ALI models. mRNA expression of IL‐6 and CXCL‐1 were measured in whole lung tissue (A, C). Measurements of MPO, IL‐6 and CXCL‐1 cytokines in BAL fluid by ELISA (B, D and E). Data are represented as mean ± SD,* n* = 6, n.s. –not significant. *P* < 0.05, **P < 0.01, ***P <0.001, ****P < 0.0001

Expression of proinflammatory cytokines IL‐6 and CXCL‐1 mRNA in lung tissue modestly increased in animals that were exposed to a single injurious stimulus (VILI, LPS or HCl alone) or in animals with HCl followed by VILI (Fig. 4A and C). Direct measurement of IL‐6 and CXCL‐1 protein in BALF via ELISA (Fig. 4 B and D) correlate with mRNA expression. Combined ALI with LPS followed by VILI showed a severe upregulation of inflammatory cytokines both in whole lung tissue and BALF (Fig. 4A–C). i.t. HCL application prior to VILI had an additive effect on cytokine upregulation both at mRNA and protein level but had overall a significantly lesser effect on cytokine production than in animals treated with LPS or LPS in combination with VILI (Fig. 4A–C). In contrast VILI after i.t. HCl application lead to a significant additive increase in cytokines (Fig. 4A–B). Although MPO levels in BALF (Fig. 4C) were comparable to the upregulation of cytokines, with again LPS followed VILI having a more pronounced effect than HCl in combination with VILI. MPO levels in BALF (Fig. 4E) are consistent with the result of IL‐6 and CXCL‐1 and demonstrated robust upregulation in mice treated with LPS alone or VILI after i.t. LPS. VILI showed an additive effect in both injury models compared to the single injury with i.t. LPS or HCl alone, with LPS+ VILI showing a much more pronounced upregulation of MPO than HCl+ VILI.

## Discussion

Murine models remain essential to study underlying mechanisms of ALI/ARDS as human patients have multiple clinical variables, which cannot be controlled. Various combined ALI models have been described previously (Yamada et al. 2000; Pfeifer et al. 2015; Tetenev et al. 2015), our goal with this study was comparing i.t LPS, respectively, HCL (simulating acid aspiration) followed by VILI. We hypothesized that exposing the lungs to an initial injury prior to inducing a secondary lung injury with VILI would lead to a superadditive effect in ALI compared to the animals only exposed to VILI.

Our animal data demonstrate that i.t LPS, respectively, HCl exposure and acid aspiration lead to modest and near equivalent disruptions in barrier function and subsequent albumin leakage in the BAL when employed separately, but when delivered in combination with VILI 24 h after initial injury, the ALI after the “second hit” has an additive effect (Fig. 2C–H). Cheng et al. (2016) in a combined injury model of hemorrhagic shock followed by cecal ligation induced sepsis also noted increased protein leakage in BAL, however whether it was additive compared to single injury cannot be assessed as only the combined injury model was discussed VILI followed after LPS exposure results in a profound upregulation of inflammatory pathways compared to animals subjected to i.t. HCl prior to VILI (Fig. 4). We speculate that priming the innate immune system of the animals with i.t. LPS prior to inducing a secondary lung injury with VILI led to a superadditive effect compared to pretreating the animals with i.t. HCl, which mostly causes direct injury to the alveolar epithelium. This is in line with the findings of Sakhatskyy et al. (2017) who reported the exposing mice to cigarette smoke prior to inducing ALI with LPS, led to exacerbation of acute lung injury and Looney et al. (2009), who showed that priming mice with LPS worsens transfusion‐induced ALI.

Mice treated with i.t. HCl and LPS showed minor fibrosis on day 3 (Fig. 2C) with a trend in the HCl animals to have increased collagen deposition. The observed fibrosis (which was confirmed also by trichrome staining in Fig. S2) is mostly around the smaller airways and is mild compared to animals exposed to a chronic inflammatory stimulus such as *chlamydia pneumoniae* infection (Jupelli et al. 2013) or i.t. bleomycin one of the classic murine models for lung fibrosis (Liu et al. 2010; Knipe et al. 2017).

This increased cytokine production is not merely a function of increased influx of inflammatory cells as the level of cellular infiltrates is similar in animals in the LPS+VILI and the HCl+VILI group (Fig. 3C and F). Of note, cellularity and MPO in BALF of the acid exposed animals (both in animals solely exposed to HCl and in combination with VILI) display a greater variability than the mice exposed to LPS, which is comparable with prior studies (Kennedy et al. 1989; Tetenev et al. 2015). Which is in line with clinical observations, that some patients will develop a mild self‐limiting pneumonitis after aspiration, whereas others progress to severe ARDS (Warner et al. 1993).

We have chosen mechanical ventilation as our second injury because of its clinical relevance. Implementation of protective ventilation with low tidal volumes into the clinical practice was the most important clinical improvement in the prevention and therapy of ARDS over the last decades (Brower et al. 2000). Despite ventilation with lower tidal volumes have been shown to reduce morbidity and mortality and multiple studies, there is still a wide variety in clinical practice and a substantial number of patients does not receive ventilation with low tidal volumes (Young et al. 2004; Needham et al. 2012, 2015; Weiss et al. 2016). Therefore, using a murine high pressure/volume ventilation model is still a clinically relevant and widely used model of ALI (Matute‐Bello et al. 2008, 2011). With the implementation of low tidal volume ventilation strategy VILI is less likely to be the sole cause of ARDS but rather a consequence of treatment. We chose to apply VILI at lower peak pressure settings than often utilized in murine VILI (Eckle et al. 2013; Hoegl et al. 2015) in order to better model a clinical situation, where human ARDS patients could still sustain VILI in addition to the initiating injury. However, we are aware that our applied peak pressure of 35 cm H_2_O is still considered high‐pressure ventilation in human patients. Further studies should incorporate additional controls in which mice are ventilated with low tidal volume after an initial pulmonary injury. This would provide valuable insight whether low tidal volume ventilation, which by itself did not cause ALI (see Fig. S1), might be injurious when combined with another injury prior to ventilation.

All our mice received 100% FiO_2_ during ventilation (both in the high and low peak inspiratory pressure). We are conscious of the fact, that most human patients do not receive 100% FiO_2_ and that oxygen toxicity might be an additional factor in our model – hyperoxia is a well established model for neonatal lung injury, although the animals tend to be exposed to hyperoxia for days not hours (Cox et al. 2015; Bao et al. 2017; Syed et al. 2017). In our model system we did not find an increase in inflammatory mediators in animals ventilated with 15 cm H_2_O for 4 h at 100% FiO_2_ versus 21% FiO_2_ (see Fig. S1). We chose to ventilate the mice with high FiO_2_ as we found that animals subjected to high peak pressure, became severely hypoxic and did not survive ventilation beyond 2 h. In order to control for the effects of hyperoxia both low and high peak inspiratory pressure group were ventilated with 100% FiO_2 ._

The pathophysiological mechanism of ALI remains poorly understood. Animal models of ALI continue to be an important tool for investigating the underlying mechanism of ALI. Large animal models of ALI, such as pig or sheep, might closer resemble human disease in terms of anatomic and physiologic similarities. However, murine models of ALI are the most widely utilized species in animal ALI models secondary to easy availability, comparatively low cost and wide availability of genetically modified animals (Matute‐Bello et al. 2008, 2011). Translation of insights gained from small animal studies to human disease is limited by the fact that murine models only partially capture the complex, multifactorial features of human ARDS. However, a recently published study comparing gene expression profiles of a variety of murine and rat models of ALI found a high degree of correlation with human data (Sweeney et al. 2017).

## Conclusions

We characterized two murine combined models of ALI‐ i.t. HCl to mimic aspiration or i.t. LPS followed by VILI. The described two‐hit models of ALI resulted in additive but differential acute lung injury, thus recapturing the clinical relevant multifactorial etiology of ALI and could be a valuable tool in translational research. Murine models remain woefully incomplete models for human disease, but incorporating models that recapitulate the multifactorial nature ARDS could provide improved insights in the underlying pathophysiology and pathways involved in ALI.

## Data Accessibility

## Supporting information




**Figure S1.** Ventilation with low pressure (15 cm H_2_O) does not induce histologic changes or cytokine production. Mice underwent tracheostomy and were kept anesthetized (sham) or were ventilated with peak pressure 15 cm H_2_O, PEEP 3 cm H_2_O, respiratory rate 80, FiO_2_ 100% or 21% for 4 h. Representative H&E stained lung sections are shown (A). IL‐6 and CXCL1 mRNA expression was determined with qPCR (B). Data are represented as mean ± SD, *n* = 4–6, n.s., not significant.
**Figure S2.** i.t. LPS and HCl causes mild peribronchial fibrosis on day 3. Representative lung sections stained with Masson's Trichrome stain to assess for lung fibrosis.
