# Supplementary figures and images for: Capturing the multifactorial nature of ARDS – “Two‐hit” approach to model murine acute lung injury
Source: Physiol Rep. 2018 Mar 29;6(6):e13648. doi: 10.14814/phy2.13648 (PMC5875538; doi:10.14814/phy2.13648)

S1

A

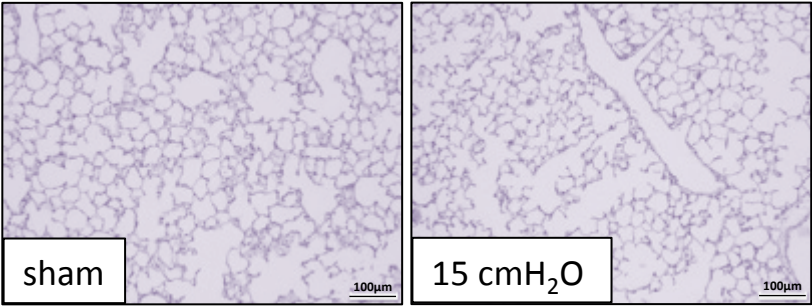

B

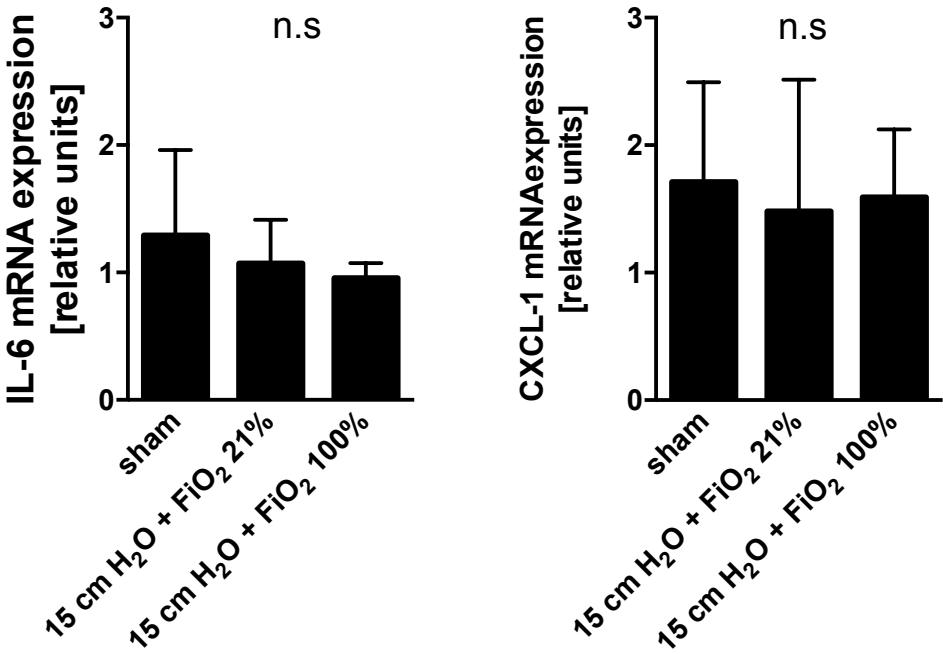

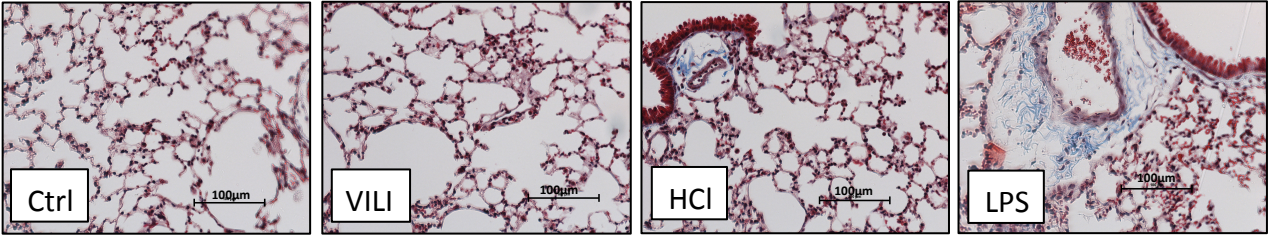

Supplement: Supplementary file 1 — Figure S1. Ventilation with low pressure (15 cm H2O) does not induce histologic changes or cytokine production. Mice underwent tracheostomy and were kept anesthetized (sham) or were ventilated with peak pressure 15 cm H2O, PEEP 3 cm H2O, respiratory rate 80, FiO2 100% or 21% for 4 h. Representative H&E stained lung sections are shown (A). IL‐6 and CXCL1 mRNA expression was determined with qPCR (B). Data are represented as mean ± SD, n = 4–6, n.s., not significant. Figure S2. i.t. LPS and HCl causes mild peribronchial fibrosis on day 3. Representative lung sections stained with Masson's Trichrome stain to assess for lung fibrosis. [file PHY2-6-e13648-s001.pdf]
