# Supplementary material for: Capturing the multifactorial nature of ARDS – “Two‐hit” approach to model murine acute lung injury
Source: Physiol Rep. 2018 Mar 29;6(6):e13648. doi: 10.14814/phy2.13648 (PMC5875538; doi:10.14814/phy2.13648)
Supplement: Supplementary file 2 [file PHY2-6-e13648-s002.docx]

**Figure S1.** Ventilation with low pressure (15 cmH_2_O) does not induce histologic changes or cytokine production. Mice underwent tracheostomy and were kept anesthetized (sham) or were ventilated with peak pressure 15 cm H_2_O, PEEP 3 cm H_2_O, respiratory rate 80, FiO_2_ 100% or 21% for 4 hours. Representative H&E stained lung sections are shown (A). IL-6 and CXCL1 mRNA expression was determined with qPCR (B). Data are represented as mean ± SD, n= 4-6, n.s. –not significant.

**Figure S2.** i.t. LPS and HCl causes mild peribronchial fibrosis on day 3. Representative lung sections stained with Masson’s Trichrome stain to assess for lung fibrosis.
